# Human otic progenitor cell models of congenital hearing loss reveal potential pathophysiologic mechanisms of Zika virus and cytomegalovirus infections

**DOI:** 10.1128/mbio.00199-24

**Published:** 2024-03-05

**Authors:** Alfred T. Harding, Karen Ocwieja, Minjin Jeong, Yichen Zhang, Valerie Leger, Nairuti Jhala, Konstantina M. Stankovic, Lee Gehrke

**Affiliations:** 1 Institute for Medical Engineering and Science, Massachusetts Institute of Technology, Cambridge, Massachusetts, USA; 2 Department of Microbiology, Harvard Medical School, Boston, Massachusetts, USA; 3 Boston Childrens’ Hospital, Boston, Massachusetts, USA; 4 Department of Otolaryngology-Head and Neck Surgery, Massachusetts Eye and Ear and Harvard Medical School, Boston, Massachusetts, USA; 5 Department of Otolaryngology-Head and Neck Surgery, Stanford University School of Medicine, Stanford, California, USA; 6 Department of Neurosurgery, Stanford University School of Medicine, Stanford, California, USA; 7 Wu Tsai Neurosciences Institute, Stanford University, Stanford, California, USA; Virginia Tech, Blacksburg, Virginia, USA

**Keywords:** human cytomegalovirus, organoid, inner ear, Zika virus, hearing loss, virus infection, model system

## Abstract

Congenital hearing loss is a common chronic condition affecting children in both developed and developing nations. Viruses correlated with congenital hearing loss include human cytomegalovirus (HCMV) and Zika virus (ZIKV), which causes congenital Zika syndrome. The mechanisms by which HCMV and ZIKV infections cause hearing loss are poorly understood. It is challenging to study human inner ear cells because they are encased in bone and also scarce as autopsy samples. Recent advances in culturing human stem cell-derived otic progenitor cells (OPCs) have allowed us herein to describe successful *in vitro* infection of OPCs with HCMV and ZIKV, and also to propose potential mechanisms by which each viral infection could affect hearing. We find that ZIKV infection rapidly and significantly induces the expression of type I interferon and interferon-stimulated genes, while OPC viability declines, at least in part, from apoptosis. In contrast, HCMV infection did not appear to upregulate interferons or cause a reduction in cell viability, and instead disrupted expression of key genes and pathways associated with inner ear development and function, including Cochlin, nerve growth factor receptor, SRY-box transcription factor 11, and transforming growth factor-beta signaling. These findings suggest that ZIKV and HCMV infections cause congenital hearing loss through distinct pathways, that is, by inducing progenitor cell death in the case of ZIKV infection, and by disruption of critical developmental pathways in the case of HCMV infection.

**IMPORTANCE:**

Congenital virus infections inflict substantial morbidity and devastating disease in neonates worldwide, and hearing loss is a common outcome. It has been difficult to study viral infections of the human hearing apparatus because it is embedded in the temporal bone of the skull. Recent technological advances permit the differentiation of otic progenitor cells (OPCs) from human-induced pluripotent stem cells. This paper is important for demonstrating that inner ear virus infections can be modeled *in vitro* using OPCs. We infected OPCs with two viruses associated with congenital hearing loss: human cytomegalovirus (HCMV), a DNA virus, or Zika virus (ZIKV), an RNA virus. An important result is that the gene expression and cytokine production profiles of HCMV/ZIKV-infected OPCs are markedly dissimilar, suggesting that mechanisms of hearing loss are also distinct. The specific molecular regulatory pathways identified in this work could suggest important targets for therapeutics.

## INTRODUCTION

Congenital virus infections continue to inflict substantial morbidity and devastating disease in neonates worldwide, and hearing loss is a consequence common to multiple such infections. Human cytomegalovirus (HCMV), also known as human herpesvirus 5, is a ubiquitous DNA virus and the most common etiologic agent of global congenital infection, affecting 0.2% to 2.5% of all live births (1). Approximately one in five babies born with congenital HCMV infection will have long-term health problems including sensorineural hearing loss (SNHL), growth retardation, microcephaly, chorioretinitis, seizures, and other neurological abnormalities (2, 3). The most prevalent outcome of congenital HCMV is SNHL, occurring in 33%–65% of symptomatic patients. Although intrauterine HCMV infection is a leading cause of SNHL, the precise pathogenic mechanism remains incompletely understood. Only a small number of histologic analyses have been reported on human inner ear samples, which have been obtained from aborted fetuses and from autopsies of infants deceased due to severe neonatal infections (4, 5).

Zika virus (ZIKV) is an arbovirus that belongs to the *Flaviviridae* family. Vertical transmission of ZIKV during pregnancy has been shown to cause severe clinical manifestations in the fetus, including neurological deficits such as microcephaly (6). In addition, case series link congenital ZIKV infection to hearing loss in newborns (7, 8). A study of hearing assessment including children congenitally exposed to ZIKV, with or without microcephaly, suggested a relationship to auditory impairment (9). Therefore, it remains unclear whether hearing loss is due to the direct effect of the virus on inner ear cells, possibly eliciting the host’s immune reaction, or due to the virus’s demonstrated ability to disrupt central nervous system development (7, 10). Neural progenitor cells infected by ZIKV have been shown to exhibit cell death (11, 12), impairment of cell cycle progression (12), premature differentiation, and defective cell division (13). However, our understanding of ZIKV-mediated pathogenesis in human SNHL is severely limited, in part because improved experimental models of the human inner ear are needed.

Human-induced pluripotent stem cells (hiPSCs) have received increasing attention as a powerful cellular platform to generate cell types of interest to understand mechanisms governing human biology, physiology, and genetics (14). The development of an efficient method to generate inner ear hair cells or auditory neurons *in vitro* using pluripotent stem cells has been the subject of intense investigation over the past decade, and several methods for inducing human otic lineage cells from hiPSCs are currently available (15–18). Two-dimensional (2D) culturing is cost-effective, permits cell observation and measurement, and is well-suited to assessing cellular host-virus interactions (19). In this study, we have derived otic progenitor cells (OPCs) from hiPSCs to study HCMV and ZIKV pathogenesis, with possible relevance to human SNHL. This human *in vitro* model of hearing loss could be useful for future development and testing of novel vaccines and therapeutics with otoprotective and otoregenerative properties.

## RESULTS

### Generation of otic progenitor cells from hiPSC

**Fig 1:**
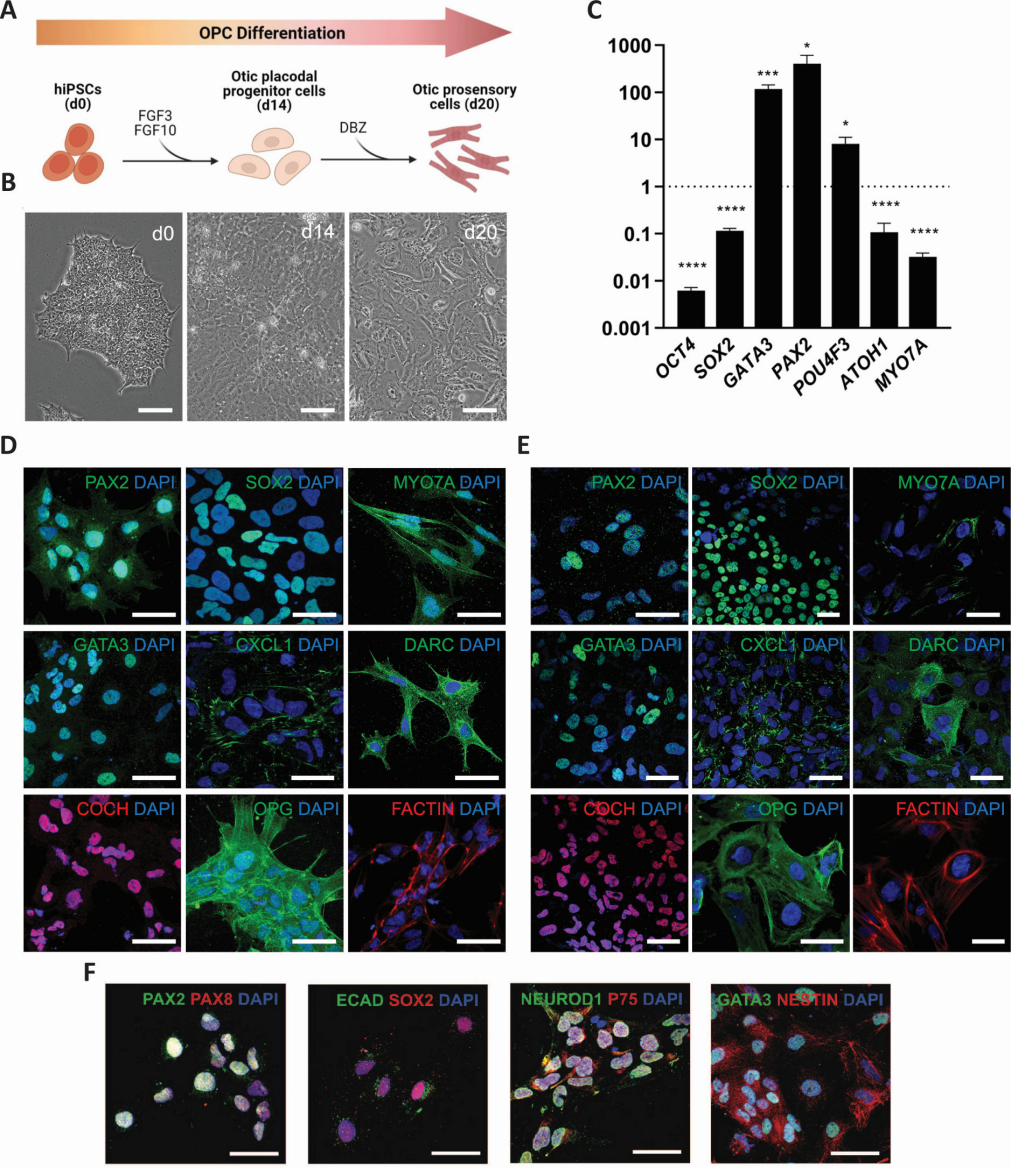
Differentiation of hiPSC into otic progenitor cells. (**A**) Schematic summary of the differentiation protocol for deriving the otic progenitor cells from hiPSCs. (**B**) A bright-field view of SK8-A hiPSCs (d0), otic placodal progenitor cells (d14), and otic progenitor cells (d20). Scale bars, 100 µm. (**C**) Quantitative reverse transcription polymerase chain reaction (RT-qPCR) data showing expression of otic-specific marker transcripts and control transcripts expressed as fold change of d20 OPCs relative to SK8-A hiPSCs (d0). Data were normalized to control for the amount of RNA isolated from SK8-A hiPSCs (d0). *n* = 3 biological samples, 3 technical repeats; *P < 0.05, ***P < 0.001, ****P < 0.0001; mean ± SEM. Panels (**D–F**) Representative images of the expression of otic lineage markers by immunocytochemistry in OPCs derived from UCSD112i-2–11 (**D, F**) and SK8-A (**E**) 20 days post differentiation. Scale bars, 50 µm. At least three biological samples and three technical repeats. Abbreviations: d, days; DBZ, difluoro-benzeneacetamide; OCT4, octamer-binding transcription factor 4; SOX2, sex-determining region Y-box 2; GATA3, GATA binding protein 3; PAX2, paired box gene 2; POU4F3, POU class 4 homeobox 3; ATOH1, atonal homolog 1; CXCL1, C-X-C motif chemokine ligand 1; DARC, duffy antigen/chemokine receptor; COCH, cochlin; OPG, osteoprotegerin.

The hiPSC SK8-A line was generated as described previously (20), and the UCSD112i-2–11 line was obtained from WiCell (Madison Wisconsin). Methods and validations for differentiating otic progenitor cells and otic lineage cells from hiPSC center on modulating fibroblast growth factor (FGF) and Notch signaling pathways as we described previously (20) (Fig. 1A). Bright-field views of d0 SK8-A cells show a typical colony formation (Fig. 1B). At d14, the morphology assumes that of otic placodal progenitor cells as characterized by the flatter monolayer. By d20, the morphology shifts toward the elongated appearance of otic progenitor cells with their ability to grow as single cells (Fig. 1B).

Quantitative RT-PCR (Fig. 1C) data showed that early otic progenitor markers GATA binding protein 3 (*GATA3*) and paired box gene 2 (*PAX2*) were highly upregulated in SK8-A otic progenitor cells compared to hiPSCs, whereas pluripotency markers such as *OCT4* (octamer-binding transcription factor 4) and *SOX2* (SRY Box 2) were downregulated. Although 20 days of differentiation is insufficient for otic progenitor cells to express key genes of hair cell development such as atonal homolog 1 and myosin VIIA (*MYO7A*), upregulation of POU class 4 homeobox 3 (*POU4F3*), which is an important inner ear hair cell marker, is consistent with the differentiation toward otic epithelial lineage.

In addition to the analysis of mRNA levels, otic progenitor cells derived from UCSD112i-2–11 (Fig. 1D and F) and SK8-A hiPSC (Fig. 1E) were identified by immunostaining with otic lineage markers. During generation of inner ear organoids from hiPSCs, otic placode/pro-sensory otic vesicles are defined by expression of transcription factors PAX2, paired box gene 8 (PAX8), SOX2, and E-cadherin (ECAD) (16). Accordingly, we observed coexpression of PAX2/PAX8 and ECAD/SOX2 (Fig. 1F), consistent with differentiation toward otic lineage rather than epibranchial placodes, optic stalk, and mid-hindbrain. MYO7A protein expression (Fig. 1D and E) also suggests that some of the cells were in the process of differentiating into hair cell-like cells. Immunofluorescence staining identified neuronal differentiation 1 (NEUROD1), GATA3, and NESTIN proteins, which are required for inner ear neurogenesis (Fig. 1F) (21). Moreover, positive expression of nerve growth factor receptor (P75 or NGFR), C-X-C motif chemokine ligand 1 (CXCL1), duffy antigen/chemokine receptor (DARC), cochlin (COCH), and osteoprotegerin (OPG) reflects the potential of otic progenitor cells to differentiate into many different inner ear cell types (Fig. 1D through F). Specifically, P75 and CXCL1 expressions were previously reported in pillar cells, while CXCL1 receptor DARC expression was reported in cochlear hair cells, supporting cells, and spiral ganglion neurons (22). COCH is abundantly expressed in the spiral limbus and spiral ligament (23), while OPG is a key regulator of bone remodeling and plays a role in function and maintenance of the auditory nerve (24). Together, these data are evidence of successful differentiation of otic lineage cells from hiPSC.

### Cytomegalovirus and Zika virus infect OPCs

To determine if HCMV and ZIKV infect iPSC-derived human OPCs, an enhanced green fluorescent protein (eGFP)-expressing HCMV, strain TB40/E-UL32-GFP (HCMV^GFP^) (25) was used to infect UCSD112i-2-11 and SK8-A cell-derived OPCs (20) at MOI 0.5 or 2.5. The corresponding ZIKV infections were performed [multiplicity of infection (MOI) 0.5] with ZIKV strain PRVABC59 (ZIKV^PR^), a 2015 Puerto Rico isolate that is associated with congenital central nervous system anomalies, including hearing loss (26, 27). Heat-inactivated virus controls were included to control for any possibility that cell host responses were due to non-specific and non-viral components of the virus inoculum.

**Fig 2:**
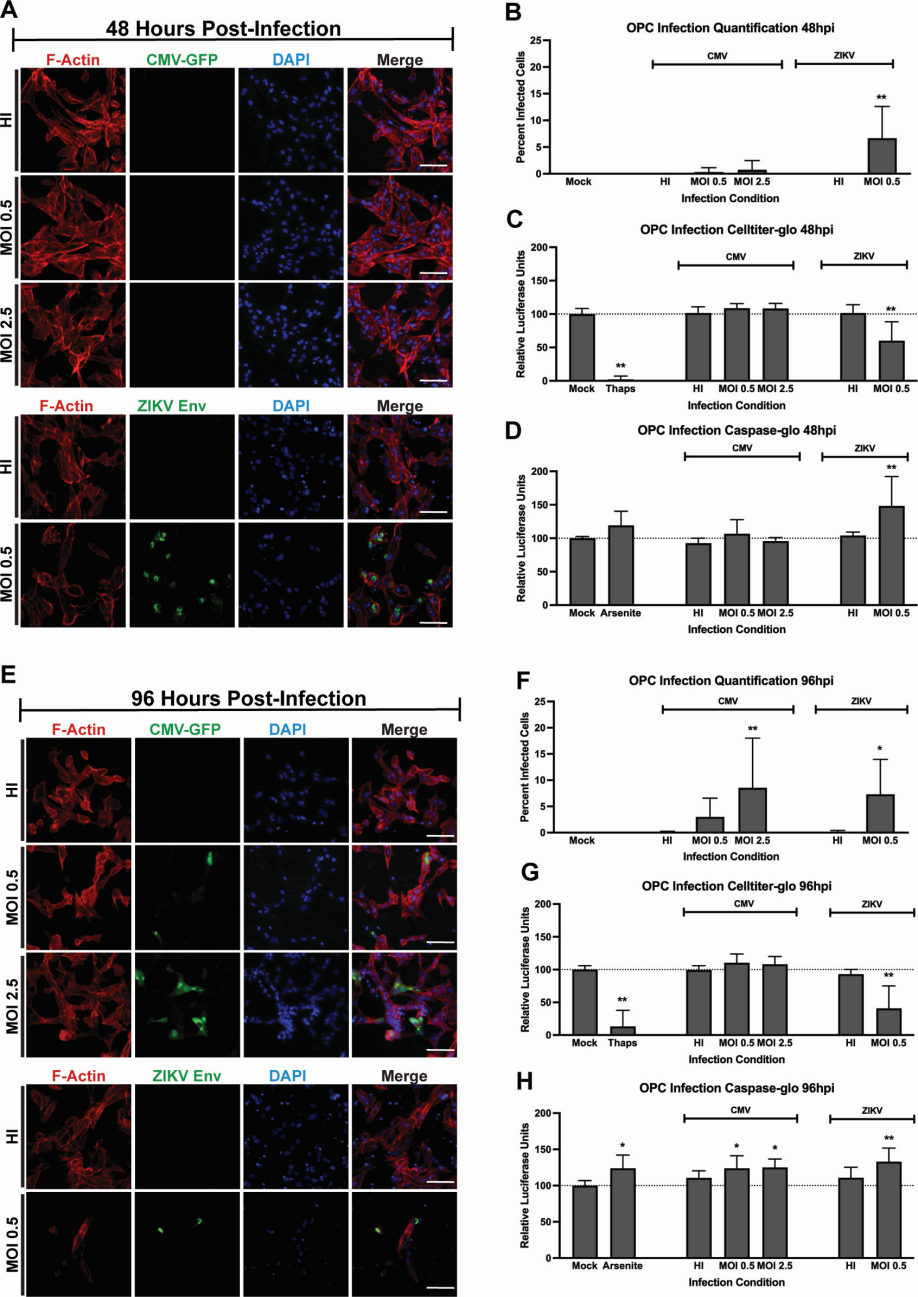
ZIKV and HCMV infect human iPSC-derived otic progenitor cells. All statistical comparisons are to mock-infected cell values. Panels A and E: imaging of OPCs infected by HCMV or ZIKV at 48 hpi and 96 hpi, respectively. X-axis labels in A–E represent imaging with (i) immunofluorescent anti-actin antibody (F-actin), (ii) GFP-tagged CMV or immunofluorescent anti-ZIKV envelope protein antibody; (iii) DAPI (4′,6-diamidino-2-phenylindole), and (iv) merge of the signals. HI, heat-inactivated virus. Panels B and F: quantification of virus-infected cells at 48 hpi and 96 hpi; panels C and G: quantification of cell viability using the CellTiter-Glo assay; panels D and H: quantification of apoptosis activity activity using the Caspase-Glo assay. All experiments are representative of two independent experiments with each OPC cell line using *n* = 3 replicates per experiment. For all Caspase and CellTiter-Glo experiments, the same number of cells were plated in each well prior to infecting, and samples were normalized based on luciferase activity of the mock condition.

Virus-infected cultures were imaged and analyzed at mid- and late stages of the replication incubations by quantifying numbers of infected cells at 48 or 96 h post-infection (hpi) (Fig. 2A, B, and F). Additional assays were included to assess cell viability and apoptosis (Fig. 2C, D, G, and H). Using an anti-ZIKV envelope antibody, or detecting the HCMV-GFP reporter signal, we first visualized the cells at 48 hpi, where we observed little detectable HCMV-GFP signal at MOI 0.5 or MOI 2.5 (Fig. 2A). ZIKV envelope signal was detected in ZIKV-infected OPCs at MOI 0.5 (Fig. 2A). We expected low-level GFP expression at 48 h because HCMV replicates more slowly than ZIKV (28). When compared to mock-infected OPCs, the HCMV-infected OPCs showed little change at 48 hpi; however, ZIKV-infected OPCs showed signs of stress as reflected by floating cells in the media, as well as fewer visible actin-stained cells (Fig. 2A). The remaining adherent cells appeared to round up, which also suggests cell stress (Fig. 2A).

In image quantification from both SK8-A and UCSD112i-2–11 cells, we observed a significant increase in infected cells, compared to mock-infected, only in the ZIKV-infected cells at 48 hpi (Fig. 2B). We next evaluated the viability of our infected OPC lines using the CellTiter-Glo assay and found that at 48 hpi, HCMV-GFP infection did not alter viability relative to mock-infected OPCs, whereas ZIKV infection significantly reduced OPC viability alongside our positive control thapsigargin, which is well known to induce cell death (29) (Fig. 2C). As it is well-documented that ZIKV infection often induces apoptosis (30), we used the Caspase-Glo 3/7 kit to determine if diminished OPC viability correlated with apoptosis. Indeed, we detected significantly elevated caspase signal in ZIKV-infected OPCs, but not HCMV-infected OPCs at 48 hpi (Fig. 2D), suggesting that at least some part of the decreased viability during ZIKV infection is due to viral-induced apoptosis. Elevated signal was also observed in the arsenite-treated positive control group; moreover, as a further control, we observed no change in caspase activity at 48 hpi when heat-inactivated (HI) virus was applied (Fig. 2D).

OPC infections were also evaluated at 96 hpi, where HCMV-GFP signal is detectable at MOI 0.5, and clearly observed at MOI 2.5, both without significant disruption of the actin staining (Fig. 2E). In the 96 hpi ZIKV infections, however, most cells had lysed or lifted off of the dish at MOI 0.5 (Fig. 2E). By quantifying the numbers of infected cells of both lines, we observed that the data trended well with initial visual observations, that is, both the MOI 2.5 HCMV-infected and MOI 0.5 ZIKV-infected cells had significant infection increases over those conducted using heat-inactivated virus (Fig. 2F). At 96 hpi, cell viability was not affected by HCMV infection at either MOI (Fig. 2G). ZIKV-infected OPCs displayed a larger decrease in CellTiter-Glo viability signal at 96 hpi, as expected, based on the large number of cells that had lifted from the dish (Fig. 2G). Caspase activity suggests that HCMV infection caused a low but statistically significant apoptosis increase, indicating that HCMV elicits a low level of host stress (Fig. 2H) in comparison with the consistently higher caspase levels associated with ZIKV infection. Moreover, after a 96-h incubation period, ZIKV infection caused OPC death that correlates with apoptosis. In comparison, the HCMV infections correlated with a mild increase in apoptosis activity, but no detectable viability change. These data are evidence that OPCs are permissive to infection by both ZIKV and HCMV; moreover, the iPSC-based model enabled the comparison of host responses elicited to HCMV and ZIKV infections, which both cause hearing loss.

### RNA sequencing (RNAseq) analysis

**Fig 3:**
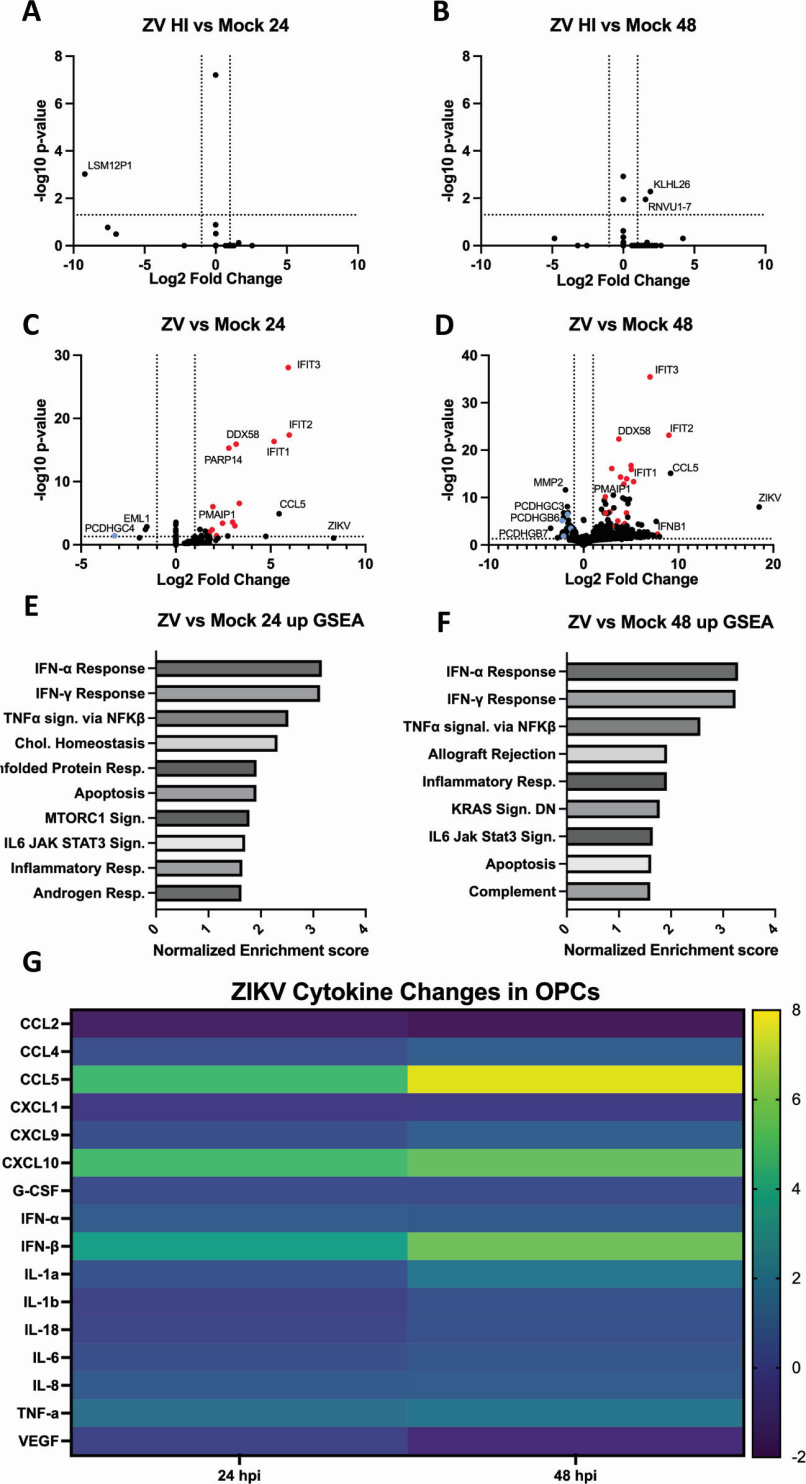
RNAseq and cytokine analysis of ZIKV-infected SK8-A and UCSD112i-2–11 OPCs. Panels A–D: volcano plots showing differentially expressed genes from OPCs infected with heat-inactivated control ZIKV at 24 and 48 hpi (**A and B**) or infectious ZIKV (**C and D**), as compared to mock-infected OPCs. Panels E and F: gene set enrichment analysis (GSEA) of ZIKV-infected OPCs at 24 hpi and 48 hpi, respectively. Panel G: Luminex cytokine analysis of supernatants from ZIKV-infected OPCs at 24 and 48 hpi. Results are displayed as a log2-fold change relative to mock-infected cells.

**Fig 4:**
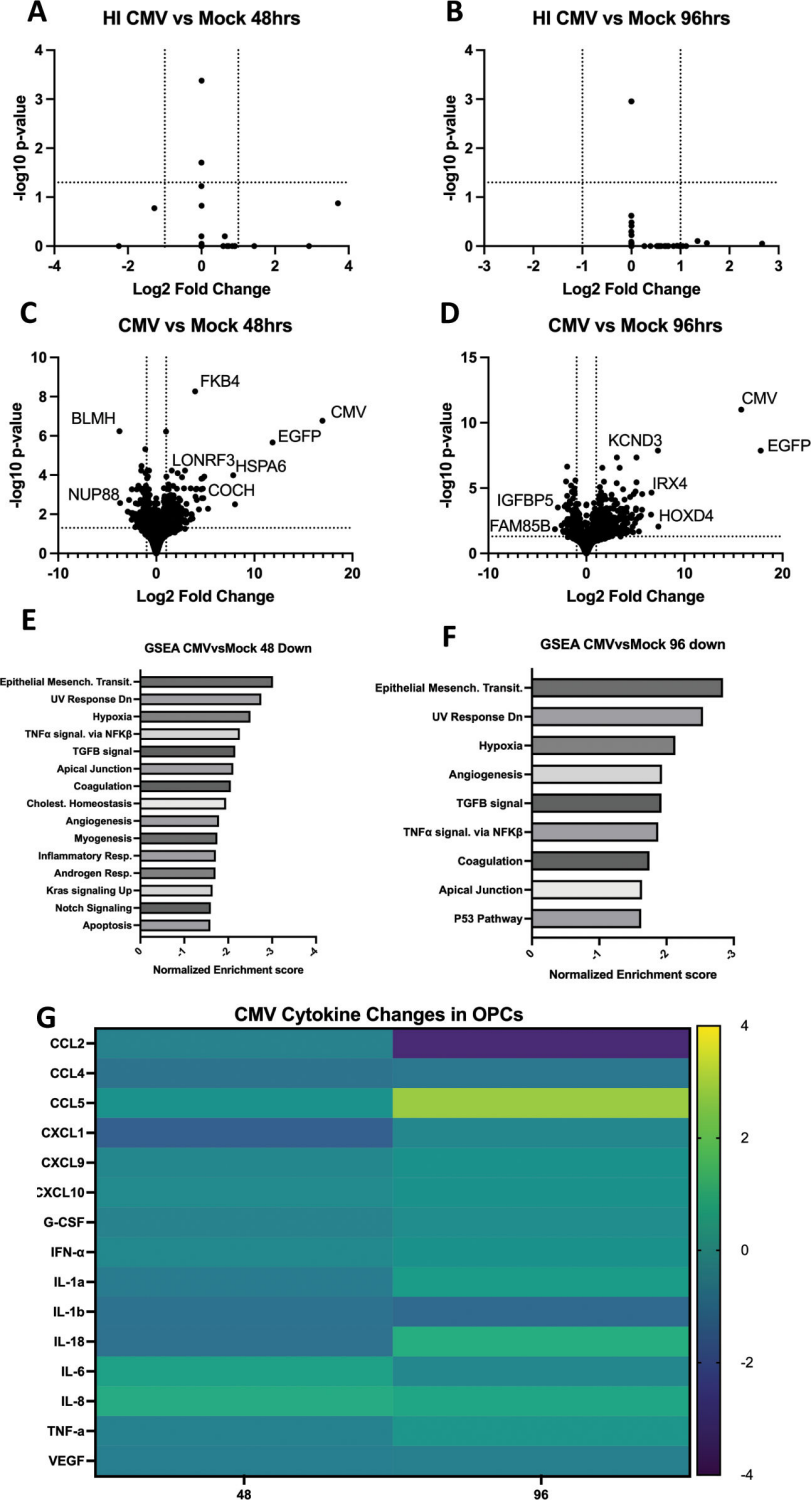
RNAseq and cytokine analysis of HCMV-infected SK8-A and UCSD112i-2–11 OPCs. Panels A–D: volcano plots showing differentially expressed genes from OPCs infected with heat-inactivated control HCMV (**A and B**) and parallel infectious CMV (**C and D**) at 48 and 96 hpi as compared to mock-infected cells. Panels E and F: gene set enrichment analysis (GSEA) of HCMV-infected OPCs at 48 hpi and 96 hpi, respectively. Panel G: Luminex cytokine analysis of supernatants from HCMV-infected OPCs at 48 and 96 hpi. Results are displayed as a log2-fold change relative to mock-infected cells.

To define and compare the impacts of ZIKV and HCMV infections on the host OPC transcriptome, RNAseq was performed using several conditions: mock infection, ZIKV infection (24 and 48 hpi), HCMV infection (48 and 96 hpi), as well as heat-inactivated virus infection, and using both hiPSC-derived cell lines. For the ZIKV RNAseq analysis, we chose 24 and 48 hpi timepoints, as suggested by data described in Fig. 2, reasoning that these times represent early and mid/late infection stages. The same reasoning was followed for the HCMV infections, except that we used 48-and 96-h timepoints because of HCMV’s longer replication period. To control for the possibility that the ZIKV or HCMV stocks contained non-viral components that non-specifically activate OPC responses, an aliquot of each stock was heat-inactivated before infecting cells (31). RNA for RNAseq analysis was extracted from all cells of a given condition, without attempting to first separate cells containing viral genomes (“infected”), from cells without viral genomes and not impacted by by virus infection (“uninfected”), and from cells without viral genomes but affected by local virus infection (“bystander cells”). This approach approximates the transcriptional environment that might be present during an *in vivo* infection. We note, however, that we cannot parse sequences from any specific cell type. Thus, all conclusions from the RNAseq data are derived from cells forming the overall environment. Using cut-off values of log2-fold change of 1 and *P*-value less than or equal to 0.05, the bulk RNAseq data revealed that only one gene at 24 hpi and two genes at 48 hpi were differentially regulated in the ZIKV heat-inactivated group (Fig. 3A and B). For HCMV, the heat inactivation experiments revealed no significant differences in the transcriptional profile at either 48 or 96 hpi (Fig. 4A and B). These data strongly suggest that the OPC responses to the HCMV and ZIKV inocula resulted from virus replication, and not due to non-specific non-viral components. The RNAseq data were analyzed as described in Materials and Methods, and a table of differentially expressed genes whose metadata referred to hearing function is presented as Table S1.

### Differentially regulated genes in Zika virus infections

Infectious ZIKV differentially activated expression of many genes (Fig. 3C and D). At 24 hpi, the majority of upregulated genes were interferon-stimulated genes (ISGs; shown as red closed circles), as expected for a response to an RNA virus infection. Importantly, among differentially regulated transcripts were several key stress/apoptosis-associated genes, including *DDIT3* (CHOP) and *PMAIP1* (NOXA) (Fig. 3C). Stress- and apoptosis-associated genes were also detected at 48 hpi, suggesting that ZIKV infection in OPCs induces a strong antiviral response that includes apoptosis-related genes (Fig. 3D). Interferon-related genes and apoptosis gene sets were also found to have high normalized enrichment scores in gene set enrichment analysis (GSEA) at both timepoints (Fig. 3E and F). GSEA also revealed ZIKV downregulated pathways at 24 and 48 h; however, identifiable trends aligned with recognizable regulatory paths were less obvious (Fig. S1).

We noted differential expression of a number of genes that are related to the inner ear and hearing (Fig. 1C and D). For example, protocadherin transcript levels were downregulated in ZIKV-infected OPCs at both 24 and 48 hpi (shown as blue closed circles). Further, *SEMA3C* and *SEMA3F*, two members of the semaphorin class 3 family of neuronal guidance genes, were also downregulated at the 48 hpi timepoint in ZIKV infection, suggesting that ZIKV infection may reduce neuronal maturation or synapse formation (Table S1). The *EML1* (echinoderm microtubule-associated protein-like 1) gene is linked to deafness and blindness in Usher syndrome type 1. The data demonstrate that at 24 h, *EML1* was significantly downregulated in ZIKV-infected OPCs (Fig. 3C; Table S1) (32). Finally, we discovered that *CXCL12*, a chemoattractant for cellular movement and angiogenesis that has been shown to be important in both ear repair and development, was also significantly downregulated 48 hpi (Table S1) (33, 34).

### Differentially regulated genes in HCMV infections

In contrast to the strong interferon response observed in ZIKV infections, HCMV infections were not characterized by a broad upregulation of interferon-stimulated and inflammatory genes (Fig. 4C and D). The absence of interferon responses in HCMV infections is perhaps consistent with earlier reports that the HCMV UL83-coded pp65, an abundant viral protein, inhibits Interferon (IFN) signaling (35). In contrast to ZIKV infection, there were no obvious patterns of upregulated apoptosis or stress-associated gene sets in the HCMV infections at either 48 and 96 hpi. The normalized enrichment scores for upregulated pathways in HCMV infection are associated with metabolism and cell growth, such as Myc and E2F targets, as reflected in the GSEA data (Fig. S2). *COCH* (coagulation factor C homology gene), which encodes an abundant cochlear extracellular matrix protein, was significantly upregulated at 48 hpi in HCMV-infected OPCs (Fig. 4A; Table S1). Cochlin mutations have been correlated with non-syndromic autosomal dominant hearing loss 9 (DFNA9) (36). Interestingly, we also observed a significant induction of *POU4F1* at 96 hpi and *NGFR/P75* at both 48 and 96 hpi, both of which have been shown to be critical for regulating the development of spiral ganglion neurons in the inner ear (22).

Although the upregulated transcripts of the HCMV-infected OPCs did not present a clear pathway similar to an interferon response, we did observe a consistent and clear signature of genes that were significantly downregulated by HCMV infection (Fig. 4E and F). GSEA results placed the epithelial-mesenchymal transition (EMT) pathway at a high normalized enrichment score (Fig. 4E and F), consistent with reports that HCMV infection blocks EMT (37). GSEA at 48 hpi also drew attention to significant negative enrichment scores for transforming growth factor-beta (TGFβ) signaling, Tumor Necrosis Factor alpha (TNFα) signaling, hypoxia, and apoptosis pathways. Reductions in the latter two pathways have also been shown in other cell types during HCMV infection (38). Also, among downregulated genes, both *IGFBP3* and *IGFBP5* (insulin growth factor binding proteins 3 and 5) were significantly downregulated in the HCMV RNAseq data at both 48 hpi and 96 hpi (Table S1). Prior evidence has demonstrated that both *IGFBP3* and *IGFBP5* play key roles in supporting the development of the cochlea during mammalian development (39). We also found that NREP expression (neuronal regeneration-related protein), which was recently shown to be expressed by regenerating chicken hair cells (40), was reduced throughout HCMV infection (Table S1). Others have demonstrated that *SOX11* is both necessary and sufficient for the development of hair cells in the inner ear (41), and the data suggest that HCMV infection reduced *SOX11* expression at both timepoints (Table S1). It was of interest to observe a downregulation of *ITGAV*, *ITGβ8*, and *TGFβ* at both 48 and 96 hpi, as well as *LRRC32* at 48 hpi, and *TGFβR1* at 96 hpi (Table S1). These genes work in concert to regulate TGFβ signaling, and it has been shown that their dysregulation can alter neurovascular development and lead to neurodegeneration (42, 43). Taken together, the RNAseq data are consistent with OPC imaging, viability, and apoptosis measures in differentiating the HCMV and ZIKV host responses.

### Cytokine expression

The RNAseq data suggested that the chemokine CCL5 (RANTES) was differentially upregulated in ZIKV-infected OPCs both at 24 and 48 hpi (Table S1). To validate the RNAseq results for CCL5 levels and to examine OPCs cytokine/chemokine expression more broadly, a Luminex bead-based immunoassay was applied (Fig. 3G and 4G). In comparing the two viral infections, CCL5, an inflammatory cytokine, was significantly elevated at both the 24 and 48 hpi timepoints for ZIKV infections, consistent with the RNAseq data. In addition, CCL5 levels were elevated at the 96-hpi timepoint in HCMV infections, albeit less than those seen during ZIKV infection. Both interferon-β and CXCL10 (IP-10), a chemoattractant molecule, were upregulated in the ZIKV infections, but not in HCMV infections. We also observed a decrease in vascular endothelial growth factor (VEGF ) protein signal over time during ZIKV infection (Fig. 3G), which trends well with data showing that both type I interferons and CXCL10 block VEGF (44, 45). CCL2 levels, reported to be associated with neuronal degeneration (46), were downregulated at both the 24- and 48-hpi timepoints during ZIKV infection (Fig. 3G), and the 96-hpi HCMV infection timepoint (Fig. 4G). Levels of interleukin-18 (IL-18), also a proinflammatory cytokine, were increased at 96 hpi in HCMV infections, while similar effects on ZIKV infections were not observed. Taken together, the data presented in Fig. 3 and 4 as well as in Table S1 suggest that although both ZIKV and HCMV infections correlate with hearing loss, their transcriptional and cytokine profiles in infected OPCs are strikingly distinct.

## DISCUSSION

The rationale for this work was to develop a model system based on human cells to experimentally analyze the cellular and molecular basis of virus infections that contribute to hearing loss. ZIKV has been used previously to infect embryonic stem cell-derived, iPSC-derived cells and organoids (47, 48), mouse inner ears (26), and chicken inner ears (49). In addition, HCMV infections have been studied using iPSC-derived organoids (50). However, a side-by-side evaluation of molecular responses to neurotropic HCMV and ZIKV infections, specifically using human iPSC-derived otic progenitor cells of the hearing apparatus, has not, to our knowledge, been described previously. Studying inner ear development and mechanisms of congenital hearing loss has long been challenging, in part because the inner ear is encased in bone, with difficult access. In addition, the lack of robust *in vitro* experimental models has been an impediment to understanding inner ear disease. Here, we have applied methods for differentiating otic progenitor cells from human iPSCs and demonstrated the utility of the experimental system by analyzing HCMV and ZIKV infections, both of which correlate with neonatal congenital hearing loss.

A major finding of this work is that the host responses to ZIKV and HCMV infections are distinct, as demonstrated by imaging, cell viability, cytokine levels, and RNAseq data. Comparing HCMV and ZIKV features and characteristics reveals common attributes including neurotropism, use of integrins as surface receptors, crossing the blood-brain barrier to reach the brain, and infections resulting in hearing loss. If infection by either virus occurs during pregnancy, both ZIKV and HCMV can also cross the placental barrier and infect the developing fetus, potentially leading to a range of symptoms including fetal microcephaly (51, 52) and hearing loss (7). Acute infections by ZIKV or HCMV are typically transient, causing febrile/flu-like disease in children and adults (6). At the same time, there are important dissimilarities separating the two viruses. ZIKV and HCMV represent different virus classifications, that is, ZIKV is a small (11 kb) unsegmented positive-sense RNA virus from the *Flaviviridae* family that replicates its genome in the cytoplasm and is transmitted primarily by mosquito vectors. HCMV is a double-stranded DNA virus from the *Herpesviridae* family; HCMV replicates in the nucleus. HCMV is the largest known human virus (236 kbp) that is transmitted sexually or through body fluids. The conclusion is that ZIKV and HCMV share little in common in terms of their structures and replication processes; however, both cause hearing loss by undefined mechanisms.

The RNAseq data provide a molecular description of regulatory pathways that are activated or suppressed in the HCMV and ZIKV infections, providing a foundation for future work to define host response mechanisms. For example, we observed downregulation of integrin αvβ8 TGFβ pathway in the HCMV data set, specifically *ITGAV* and *ITGAB8*, whose protein products are reported to activate latent TGFβ (53). These genes may be relevant to understanding virus-mediated hearing loss because the TGFβ signaling pathway has been reported to be key in regulating programmed senescence in inner ear morphogenesis in both murine and chicken models (54, 55). It follows, therefore, that decreased *ITGAV* and *ITGAB8* transcripts and their translated proteins may cause developmental disorders. Further potential relevance to hearing loss mechanisms is that the absence of the *ITGAV* and *ITGAB8* genes has also been shown to cause neurological complications, including disruption of neurovascular development and neurodegenerative disease in murine models (56, 57). RNAseq data also demonstrated that the *GARP* gene (glycoprotein A repetitions predomain, alternately referred to as *LRRC32*) was significantly downregulated in the HCMV infection data set. GARP orients TGFβ for binding and activation by integrin αVβ8 (58), functioning in TGFβ signaling, which as noted above may impact inner ear morphogenesis. Finally, we also noted that COCH transcripts, encoding the cochlin protein, were significantly upregulated by HCMV infection (Fig. 4). Cochlin is an extracellular matrix protein that is abundant in the inner ear, comprising up to 70% of inner ear protein in bovine models. Mutations of *COCH* are associated with inherited progressive sensorineural hearing loss (DFNA9) (59, 60). Cochlin overexpression has been linked to nerve damage (61); suggesting a mechanism by which the COCH upregulation observed in our data may correlate with nerve damage related to hearing loss. Taken together, these data suggest that HCMV infections result in dysregulation of critical genes that control the development of the inner ear, resulting in hearing loss.

During ZIKV infections, we discovered a very different molecular host response profile, as compared to HCMV (Table S1). The most notable host response was type I interferon signaling wherein we observed induction of numerous ISGs at 24 and 48 h. In addition, we identified upregulation of the interferon-β gene at 48 hpi, consistent with the interferon response typically associated with RNA virus infections. Strong IFN signaling responses observed in acute viral infection are often accompanied by induction of genes associated with cell stress (*DDIT3/CHOP*), apoptosis (PMAIP1/Noxa), and general inflammatory chemokines (CXCL10, CXCL12, and CCL5). The correlations among the RNAseq, chemokine, and cell viability assays were strong, supporting the rigor of the data. In contrast to the HCMV data, the results suggest that ZIKV infection causes hearing loss by inducing cell stress/death of infected cells.

Although both HCMV disease and ZIKV congenital disease affect hearing, little was known, prior to this study, about whether the hearing loss was based on similar or different pathogenesis mechanisms. To address these questions, we used cells derived from human iPSC. A distinguishing feature of our approach is the side-by-side virus infections, using common virus stocks and cells to define differential host responses. We demonstrated that the OPCs were infected by both ZIKV and HCMV, a first step in the development of viral pathogenesis. The data show that the host responses to HCMV and ZIKV infections were not only different but are seemingly opposite at the molecular level. ZIKV was found to activate both interferon responses and apoptotic signaling pathways that correlate with rapid destruction of OPCs. In contrast, HCMV-infected cells remain viable but appear to have dysregulated expression of key inner ear developmental genes/pathways. These differences suggest divergent mechanisms underlying ZIKV- and HCMV-associated congenital hearing loss. ZIKV may interfere with the development of the inner ear by killing the progenitor cells, while the effects of HCMV infection may instead stem from blocking normal developmental pathways. An implication of the work described here is that strategies to prevent and treat congenital ZIKV and HCMV hearing loss must consider the marked differences in host responses to viral infections. The hIiPSC-derived OPC model serves as a bridge between animal studies and humans, and may have an impact on encouraging additional research to define the molecular mechanisms of viral pathogenesis affecting the inner ear.

## MATERIALS AND METHODS

### Generation of hiPSC line SK8-A

SK8-A cells were generated as described previously (20) from healthy donor fibroblasts using Sendai virus transduction of the reprogramming factors. Approximately 3 weeks after viral transduction, colonies with stem cell morphologic characteristics appeared and were clonally expanded on mouse embryo fibroblasts. Markers for successful reprogramming into hiPSC, including normal karyotyping, immunoreactivity for embryonic stem cell-associated antigens including OCT4, NANOG, stage-specific embryonic antigen (SSEA4), and T cell receptor alpha locus (TRA-1-60), and the presence of self-renewal gene products [*OCT4*, *NANOG*, sex-determining region Y-box 2 (*SOX2*), DNA methyltransferase 3 alpha (*DNMT3*), and ZFP42 zinc finger protein (*REX1*)] were demonstrated previously (20). On spontaneous differentiation, the hiPSC embryoid bodies showed upregulation of lineage markers representative of the three embryonic germ layers, ectoderm [engrailed homeobox 1 (*EN1*), microtubule-associated protein 2 (*MAP2*), and nuclear receptor subfamily 2 group f member 2 (*NR2F2*)], mesoderm [snail family transcriptional repressor 2 (*SNAIL2*), a regulator of g protein signaling 4 (*RGS4*), heart- and neural crest derivatives-expressed protein 2 (*HAND2*)], and endoderm (somatostatin (*SST*), kruppel-like factor 5 (*KLF5*), aggregation-promoting factor (*APF*)], confirming differentiation potential (20).

### Zika virus

The ZIKV^PR^ strain, PRVABC59, was obtained from BEI resources and propagated in C6/36 mosquito cells in serum-free neuro-glial differentiation media (62). This protocol was adapted from standard techniques (63) for infections in neural models and produces high titer virus. Virus-containing cell supernatants were harvested after the appearance of cytopathic effect in the C6/36 cells and clarified by centrifugation. Zika-conditioned media was collected from uninfected C6-36 cells grown in parallel. Viral titer was determined by focus-forming assay to calculate BHK-21 focus-forming units: BHK-21 cells (ATCC) were infected with dilutions of viral stock and overlaid with 3.2% carboxymethylcellulose solution mixed 1:1 with Dulbeccos modified Eagle medium (DMEM) containing 2% Fetal Bovine Serum (FBS) (63). At 4 dpi, cells were fixed and stained with 5 ug/mL anti-ZIKV NS1 protein monoclonal antibody #110 (64), followed by secondary staining with Horseradish peroxidase (HRP)-conjugated goat-anti-mouse antibody (Promega) and detection using the Vector VIP HRP substrate kit (Vector Laboratories).

### Cytomegalovirus

TB40/e-UL32-GFP HCMV was a gift from Dr. Donald Coen, Harvard Medical School. Virus was propagated on human foreskin fibroblasts (HFF) (ATCC). Virus-containing supernatant was harvested after the appearance of cytopathic effect, and virus was concentrated and purified by ultracentrifugation through a 20% sucrose cushion at 86,700 g for 1 h at 4°C in polypropylene tubes (Polyallomer Beckman 331372). We found it necessary to purify HCMV by ultracentrifugation through a sucrose cushion to avoid non-viral, non-specific activation by the virus stock inoculum. The virus pellet was resuspended in DMEM 2% FBS. Mock infections were performed in DMEM. Viral titer was determined by infecting fresh HFF cells, followed by quantitative flow cytometry analysis of GFP-bright cells, to calculate HFF infectious units.

### Heat inactivation of virus stocks

Virus inactivations were performed to control for the possibility that HCMV and ZIKV stocks contained components that non-specifically activated the OPCs. Zika virus was heat-inactivated by incubation at 60°C for 1 h. HCMV was purified by ultracentrifugation through a sucrose cushion, followed by resuspension and titering. An aliquot of the resuspended and titered HCMV was then heat-inactivated by incubation at 63°C for 30 min (65).

### Virus infections

OPC virus infections were performed at multiplicities of infection calculated using BHK-21 infectious units for ZIKV^PR^ and HFF infectious units for HCMV^eGFP^. Virus was added to cells in minimal volume and incubated with intermittent rocking for 1 h at 37°C. Inoculum was then removed and replaced with fresh medium.

### Viability and apoptosis assays

OPCs were grown and infected in 96-well plates at a density of 2.6e04 cells per well. As a positive control for apoptosis-induced cell death, cells were treated with 20 µM arsenite or 20 µM thapsigargin for a 24-h period prior to each harvest timepoint. At indicated timepoints, cell viability was measured in duplicate or triplicate using the CellTiter-Glo kit (Promega), following the manufacturer’s protocol. Luminescence was measured in triplicate with a Berthold plate reader. Apoptosis was measured in triplicate using Caspase-Glo 3/7 Assay kit (Promega) following the manufacturer’s protocol. Independently differentiated OPCs were used for biological replicates.

### Luminex cytokine assays

OPCs were grown and infected in 12- or 24-well plates at a density of 3.0e05 or 2.0e05 cells per well, respectively. For all experiments, media was changed at 48 hpi unless otherwise indicated, and supernatant was harvested at indicated timepoints. Therefore, cytokines detected at 24 hpi represent cumulative production from 0 to 24 hpi, at 48 hpi represent cumulative production from 0 to 48 hpi, and at 96 hpi represent cumulative production from 48 to 96 hpi. Experiments were performed on supernatants from mock-infected, ZIKV- or heat-inactivated ZIKV-infected (MOI 0.5), HCMV, or HCMV-infected (MOI 2.5) cells. Data represent the average of samples from both SK8-A and UCSD112i-2–11 cells, which were conducted independently. Luminex assays were performed using the Human Magnetic Luminex Assay, R&D LXSAHM-16 assay targeting CCL2, CCL4, CCL5, CXCL1, CXCL9, CXCL10, G-CSF, IFN-α, IL-1α, IL-1β, IL-18, IL-6, IL-8, TNF-α, and VEGF. The Luminex assay was performed according to the manufacturer’s protocols using a Luminex MAGPIX.

### Immunofluorescence microscopy

OPCs were grown and infected in 96-well black-walled optical bottom plates. Cells were fixed at times indicated in 3.2% paraformaldehyde (PFA) for 20 min at room temperature and stored in Phosphate-buffered saline (PBS). Fixed cells were blocked in 3% normal donkey serum in 0.3% Triton X. Primary staining for ZIKV-infected cells was performed overnight using polyclonal rabbit-anti-ZIKV Envelope protein antibody (Genetex) in 3% normal donkey serum in 0.1% Triton X. Phycoerythrin conjugated goat-anti-rabbit antibody was used in secondary staining of ZIKV-infected cells, in combination with direct staining of F-actin with AlexaFluor 488 phalloidin (ActinGreen 488 ReadyProbe, Thermo Fisher Scientific) and nuclear staining by DAPI, for 1 h at room temperature. HCMV-infected cells express green fluorescent protein, and actin was stained with AlexaFluor 555 phalloidin (ActinRed 555 ReadyProbe, Thermo Fisher Scientific) and DAPI. All cells were imaged using an EVOS fluorescence microscope. One to three independent fields of cells were imaged for each condition in each experiment. Experiments were repeated in triplicate, each in independently differentiated OPCs. To determine percent infected cells, counts of virally infected cells were divided by counts of total nuclei in each field, both calculated using standard tools in ImageJ (NIH).

### Statistical analysis

The Bulk-RNA sequencing statistical analysis is described in the next section. All other statistical analyses were performed using Prism software (GraphPad Software). For these panels (Fig. 1C, 2B through D and F through H), an unpaired Student’s *t*-test was used to compare the statistical significance over mock/control conditions.

### RNAseq

RNA was extracted from cells using the Qiagen RNeasy minikit according to standard protocols. Samples were then submitted to the MIT BioMicrocenter and total RNA was sequenced using the paired end Illumina NovaSeq 6000 platform. RNAseq data were analyzed using the hg38 human assembly with the Ensembl version 106 annotation using Salmon version 1.6.0 (66). The salmon target included the Zika (KX087101.3) and the CMV (KX544839.1) genomes. Gene-level summaries were prepared using tximport version 1.24.0 (67) running under R version 4.2.1 (68). Differential expression analysis was done with DESeq2 version 1.36.0 (69, 70) and differentially expressed genes were defined as those having an absolute apeglm (71) log2-fold change greater than 1 and an adjusted *P*-value less than 0.05. Preranked gene set enrichment analysis (72) was done using javaGSEA version 4.3.2 with msigDb version v2022.1 (73) gene sets.

## Data Availability

The RNAseq data are available on the GEO Accession Database under accession number GSE234062.

## SUPPLEMENTAL MATERIAL

Figure S1 mbio.00199-24-s0001.tif ZIKV downregulated GSEA.

Figure S2 mbio.00199-24-s0002.tif CMV upregulated GSEA.

Legends mbio.00199-24-s0003.docx Supplemental material legends.

Table S1 mbio.00199-24-s0004.tif List of hearing-related genes that were perturbed by virus infection.

ASM does not own the copyrights to Supplemental Material that may be linked to, or accessed through, an article. The authors have granted ASM a non-exclusive, world-wide license to publish the Supplemental Material files. Please contact the corresponding author directly for reuse.
