# Supplementary figures and images for: Human otic progenitor cell models of congenital hearing loss reveal potential pathophysiologic mechanisms of Zika virus and cytomegalovirus infections
Source: mBio. 2024 Mar 5;15(4):e00199-24. doi: 10.1128/mbio.00199-24 (PMC11005345; doi:10.1128/mbio.00199-24)

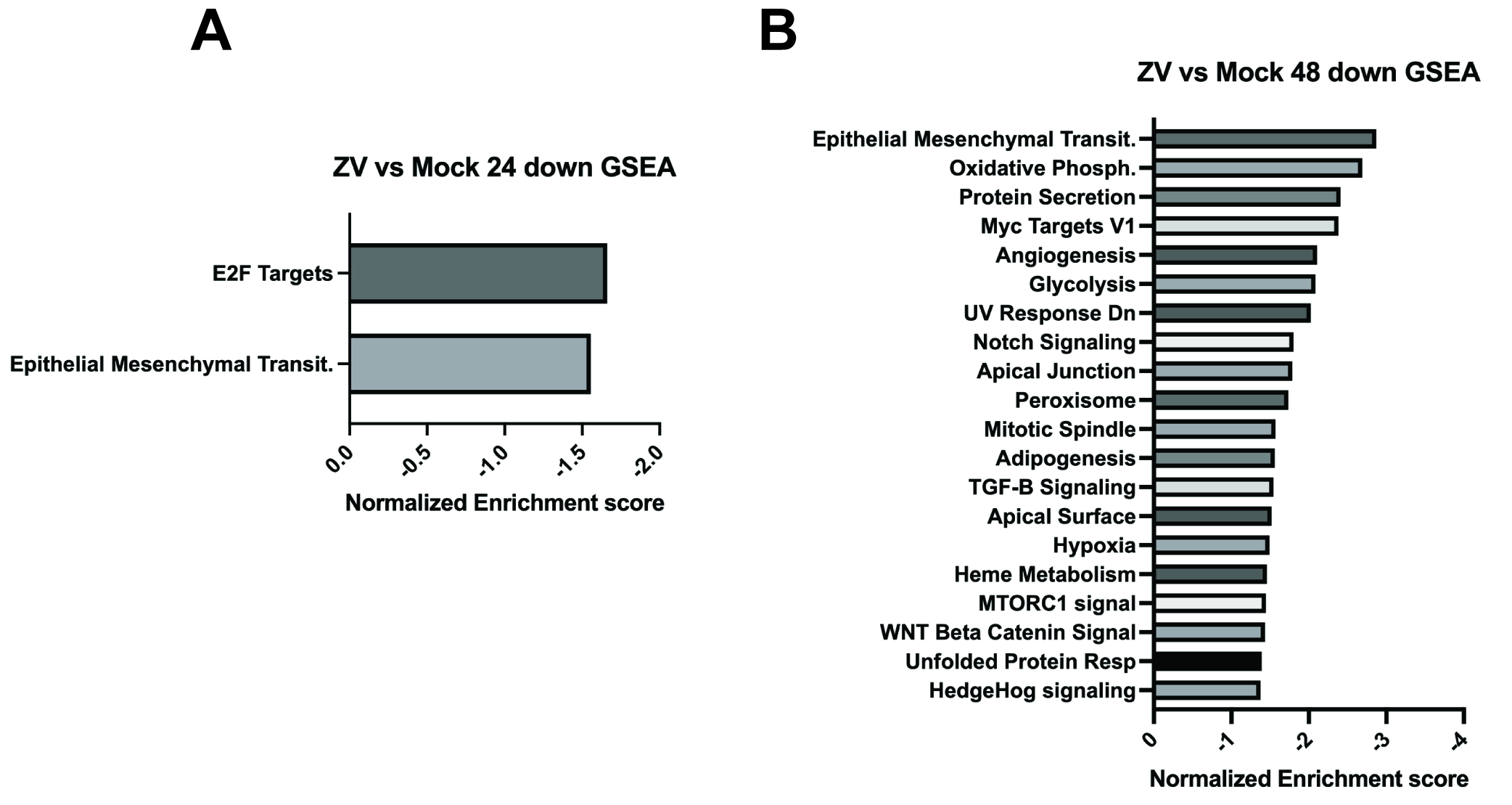

Supplement: Figure S1 — ZIKV downregulated GSEA. [file mbio.00199-24-s0001.tif]

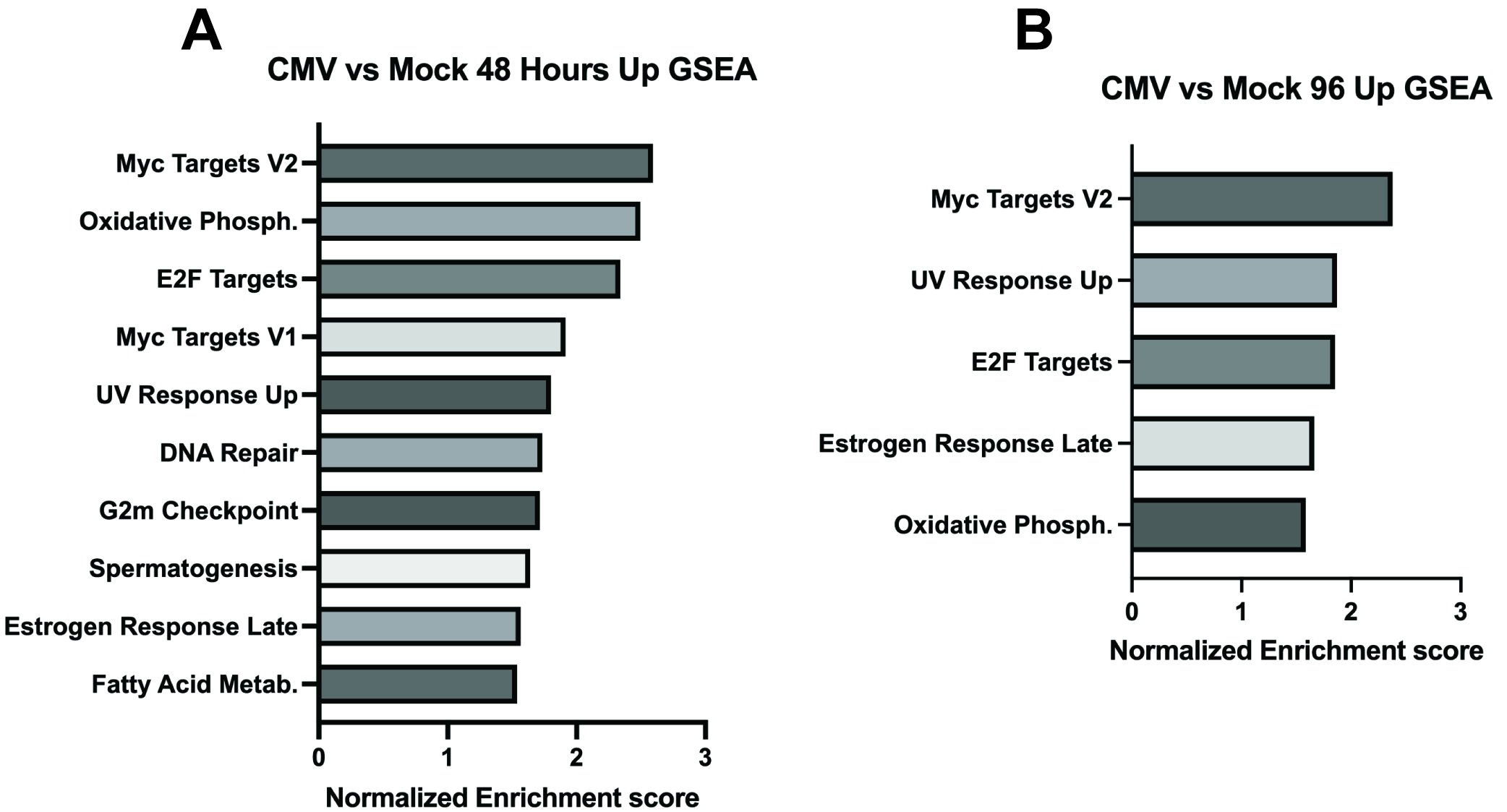

Supplement: Figure S2 — CMV upregulated GSEA. [file mbio.00199-24-s0002.tif]

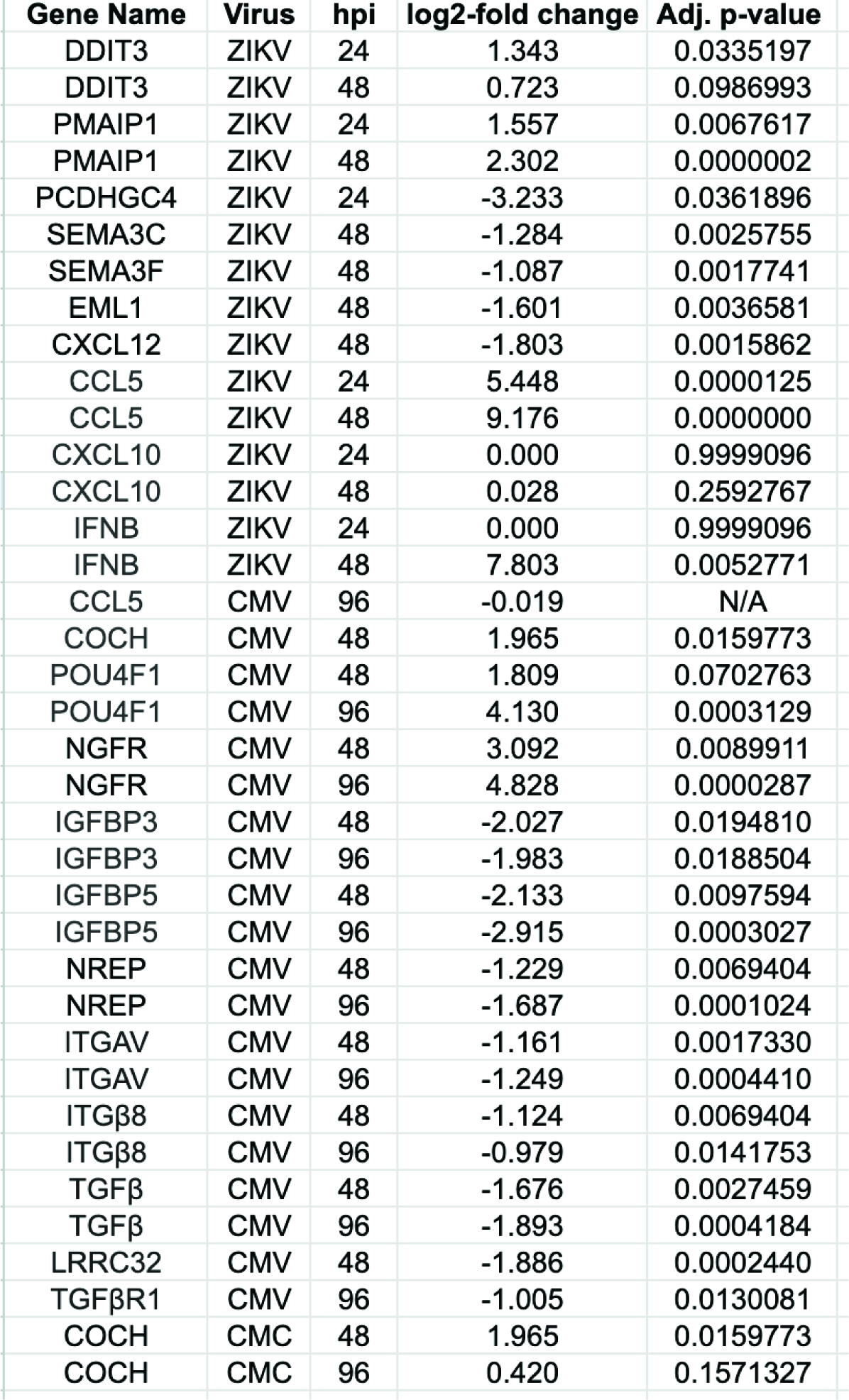

Supplement: Table S1 — List of hearing-related genes that were perturbed by virus infection. [file mbio.00199-24-s0004.tif]
