# Supplementary material for: Human otic progenitor cell models of congenital hearing loss reveal potential pathophysiologic mechanisms of Zika virus and cytomegalovirus infections
Source: mBio. 2024 Mar 5;15(4):e00199-24. doi: 10.1128/mbio.00199-24 (PMC11005345; doi:10.1128/mbio.00199-24)
Supplement: Legends — Supplemental material legends. [file mbio.00199-24-s0003.docx]

Figure S1. Downregulated GSEA Pathways in OPCs during ZIKV infection. Panels **A-B**: Pathways downregulated at 24 and 48 hpi, respectively. All pathways shown have a p-value of < 0.05. EMT: epithelial-mesenchymal transition.

Figure S2. Upregulated GSEA pathways in HCMV-infected OPCs. **A-B**: Pathways upregulated at 48 and 96 hours post-infection, respectively. All pathways shown have a p-value less than or equal to 0.05.

Table S1. List of RNAseq differentially regulated genes with hearing or inner-ear metadata, as described in the text. The fold change and adjusted p-values are shown for each gene under conditions of OPC infection by either ZIKV or HCMV at 24, 48, 96 hours post infection.
